# Electron-Induced Fragmentation of 5-Iodouridine: Implications for Enhanced Radiotherapy

**DOI:** 10.1021/acs.jpclett.5c01615

**Published:** 2025-10-03

**Authors:** Janina Kopyra, Paulina Wierzbicka, Hassan Abdoul-Carime

**Affiliations:** † Faculty of Sciences, Siedlce University, 3 Maja 54, 08-110 Siedlce, Poland; ‡ Universite Claude Bernard Lyon 1, CNRS/IN2P3, UMR5822, Institut de Physique des 2 Infinis F-69003 Lyon, Villeurbanne France

## Abstract

5-Iodouridine is a known and potentially efficient radiosensitizer; however, it has not been considered for clinical use because of its poor metabolic incorporation into DNA. Recent development of a novel pro-drug, ropidoxuridine, has improved the bioavailability of this halogenated nucleoside, although the exact mechanism of its radiosensitizing action remains not fully elucidated. Here, we demonstrate that low-energy electrons—abundantly generated along radiation tracks—efficiently dissociate the halogenated nucleoside via the primary pathway (99%), producing an iodine anion and a uridine-yl^•^ neutral radical, with a high approximate DEA cross section of (2.7 ± 1.9)×10^–14^ cm^2^. The latter, known to be highly reactive, subsequently induces hydrogen abstraction, leading to DNA strand breaks. The damage induced in 5IUrd by low-energy electrons is found to be about 700 times greater than that in thymidine and about 4 times that of the clinically used 5-fluorouridine. These findings may contribute to the development of future cancer therapy strategies by synergistically combining 5IUrd with cisplatin or gold nanoparticles, which act as a source of secondary low-energy electrons during radiation therapy.

## Graphical abstract

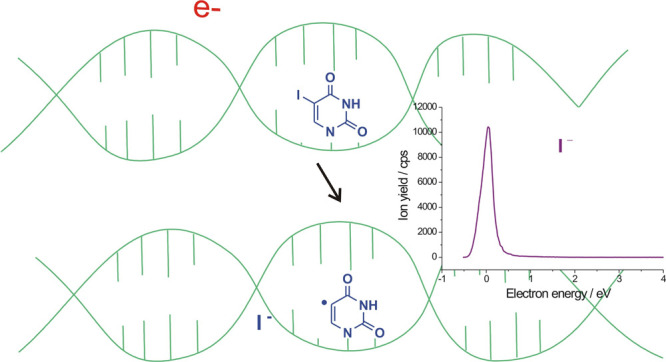

The American Cancer Society, by estimating and compiling the number of cancer cases and annual deaths, has shown that in 2024, there was a decline in cancer mortality due to several factors, including earlier detection for some cancers and improvements in treatments.^1^ Nonetheless, the progression of cancers such as breast, pancreatic, liver, colorectal or cervical cancers^1^ urges the development of more efficient and synergistic strategies with less invasive and collateral effects such as the combination of low-dose radiation^2,3^ or targeted^4^ therapies with radiosensitizing molecules.^5,6^

5-Halouracil radiosensitizers belong to the family of the canonical thymine DNA nucleobase for which the methyl group (CH_3_) at the C5-position is surrogated by a halogen atom, i.e., F, Cl, Br or I. Their use as a chemo-radiotherapeutic agent was suggested in the early 1970s, after an increased sensitivity of cells to X-ray irradiation was observed, in which a certain percentage of the thymine nucleobase was replaced by a halogenated surrogate.^7^ However, to date, 5-fluorouracil (e.g., Xeloda ) is the only molecule clinically used in radiotherapy for the treatment of several cancers.^8^ 5-Iodouridine (5IUrd) has also shown to be a radiotherapeutic agent with high potentiality.^7,9^ Unfortunately, the clinical development of this agent has been found to be limited by poor metabolic incorporation into DNA. Recently, it has been shown that the process of thymine surrogation by 5-iodouracil can be significantly improved through a synergistic association of pro-drugs,^10^ particularly ropidoxuridine (5-iodo-2-pyrimidinone), which can be converted into 5IdUrd by the aldehyde oxidase enzyme with minimal toxicity.^11^ Thus, ropidoxuridine becomes very promising for the oral treatment of a large variety of cancers, such as glioblastoma, gastrointestinal cancers (esophagus, liver, stomach, pancreas colon or rectum).^12–14^ The use of 5-iodouracil regains attractiveness for radiotherapy, and this pro-drug is now in phase II clinical trial for treatment of patients with glioblastoma.^15,16^

While the exact mechanism by which the radiosensitization with 5IdUrd occurs is not clearly identified, X-ray radiolysis experiments of double-stranded DNA, where halogenated substitution occurs in only one strand, have shown that mobile reactive intermediates cause damage to the unsubstituted strand, resulting in DNA double-strand breaks, increasing the cytotoxicity of the irradiation.^17^ For a longtime, radiosensitization of modified nucleic acids has been presumed to result from the genotoxic action of solvated electrons produced, i.e. within a time frame of microseconds after exposure to ionizing radiation.^18^ In contrast, nascent electrons present within a short time window (∼fs-ps) after the deposition of energy from the primary radiation^19,20^ can immediately induce damage to its chemical environment. Indeed, these generated presolvated electrons have an energy distribution well below 20 eV, peaking around 10 eV and mostly <0.5 eV,^21,22^ and slow down from inelastic scattering until being trapped as solvated electrons. These nascent electrons are capable of damaging DNA, causing strand breaks^23,24^ by fragmenting its constituent, particularly via the rupture of the N-glycosidic bond between the nucleobase (Nb) and the sugar moiety.^25,26^ Therefore, low energy electrons may be strongly involved in the radiosensitization of DNA by 5-halouracils.^27–29^ The present work aims to elucidate and quantify the mechanism of DNA sensitization by 5IdUrd. The findings are compared to the results obtained from the decomposition of thymidine^25^ and 5-fluorouridine^30^ by low-energy electrons.

We performed collision experiments of monoenergetic electrons with 5-iodouridine molecules in a crossed-beam arrangement,^31^ consisting of an electron source, an oven, and a quadrupole mass analyzer. The components are housed in a UHV chamber at a base pressure of approximately 2 × 10^–8^ mbar. A well-defined electron beam, generated by a trochoidal electron monochromator (operating resolution ≈ 210 meV fwhm), orthogonally intersects an effusive molecular beam of 5IUrd. This molecular beam emanates from a vessel containing 5IUrd in the form of a 99% purity powder (a product from Fluorochem). The sample, used as delivered, is loaded into the oven under an ambient atmosphere, which is then transferred into the high-vacuum chamber. During the measurements, heating lamps ensure the thermal evaporation of the solid and maintain all electrostatic lenses and plates at the oven temperature to prevent 5IUrd deposition, which could otherwise lead to undesirable changes in contact potentials. In these experiments, the vessel is heated to a maximum of 428–430 K, well below the decomposition temperature.^32^ The negative ions produced in the reaction zone after the electron-molecule interaction are extracted from the collision area by a small draw-out field (≈ 0.5 Vcm^–1^), analyzed by the quadrupole mass analyzer, and detected by a single-pulse counting technique. The electron energy scale is calibrated by using a flow of SF_6_ gas through the oven yielding the well-known SF_6_^–^ resonance near 0 eV. However, the measurements are performed without the presence of the calibration gas to avoid potentially unwanted reactions, such as dissociative electron transfer with the investigated molecules, which could produce an additional signal near 0 eV.^33^

Collision of low energy (<12 eV) electrons with 5IUrd (or M) molecules results in the formation of fragment anions shown in Figure 1 (*m*/*z* 127, 110, and 81) and Figure 2 (*m*/*z* 237, 94, 71, 46, 42, 26, and 16). The first set of species, with *m*/*z* = 127, 110, and 81, represents the most intense anion yields recorded. From the stoichiometry, they can be tentatively ascribed to I^–^, [5IUrd – I – R]^−^ (here and throughout this work, R denotes [ribose – OH]), and [5IUrd – R – I – COH]^−^, respectively. Other fragments (Figure 2), with the *m*/*z* 237, 94, 71, 46, 42, 26, and 16, are observed with considerable lower intensity and have been attributed to [5 IU – H]^−^, [5IUrd – R – I – O]^−^, [R – C_2_O_2_H_6_]^−^, [C_2_OH_6_]^−^, OCN^–^, CN^–^, and O^–^ anions, respectively. Table 1 summarizes all recorded fragment anions. The anion yields plotted as a function of the energy of the colliding electrons present a remarkable feature that is structures reminiscent of resonant processes (Figures 1 and 2).

**Figure 1:**
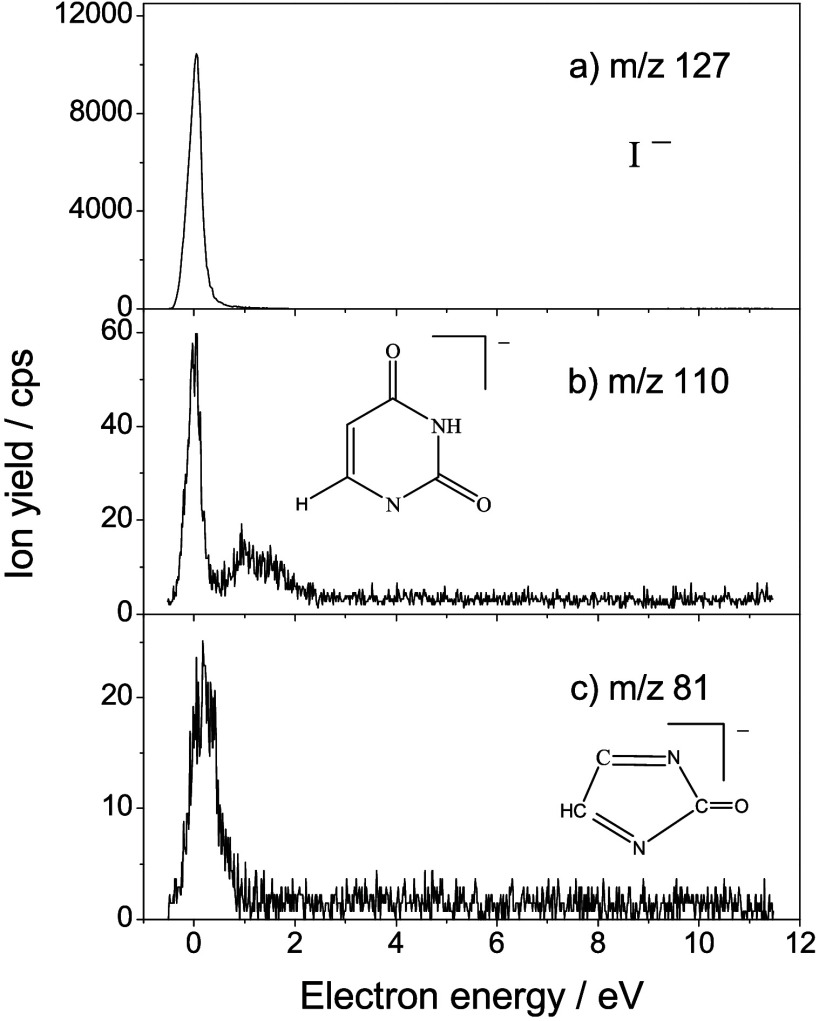
Ion yield curves for the DEA to 5-iodouridine resulting in the formation of fragment anions at a) *m*/*z* 127 (I^–^), b) *m*/*z* 110 ([5IUrd – I – R]^−^), and c) *m*/*z* 81 ([5IUrd – R – I – COH]^−^).

**Figure 2:**
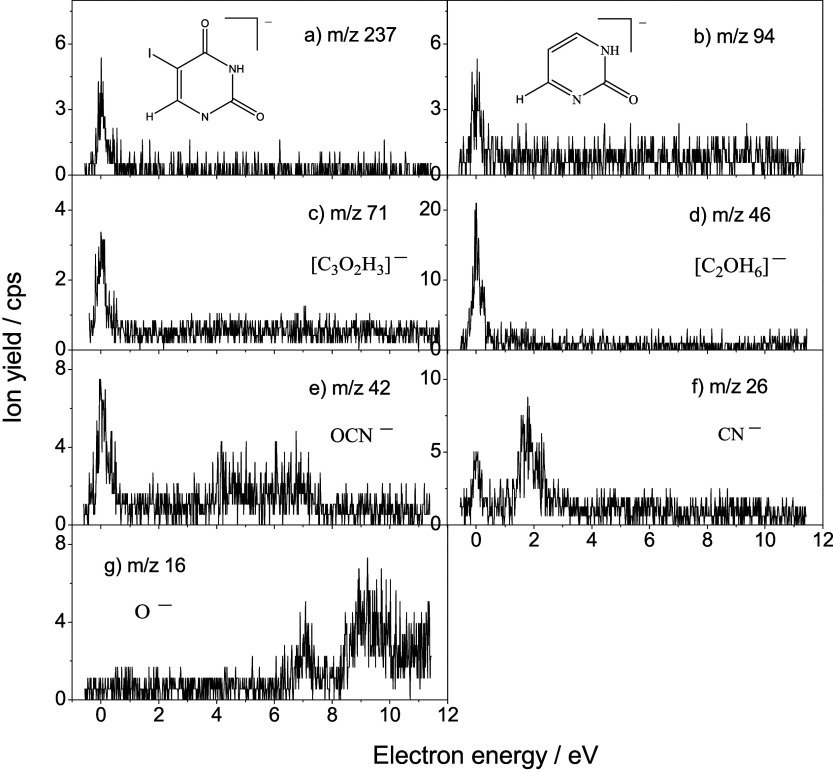
Ion yield curves for the DEA to 5-iodouridine resulting in the formation of fragment anions at a) *m*/*z* 237 ([5 IU – H]^−^), b) *m*/*z* 94 ([5IUrd – R – I – O]^−^), c) *m*/*z* 71 ([R – C_2_O_2_H_6_]^−^), d) *m*/*z* 46 (C_2_OH_6_^–^), e) *m*/*z* 42 (OCN^–^), f) *m*/*z* 26 (CN^–^), and *m*/*z* 16 (O^–^).

**Table 1:** List of Observed Anion Fragments, Peak Position (eV) and Tentative Assessment of the Species and Their Origins^a^

*m*/*z*	Peak maximum (eV)	Anion fragment assessment (origin of the fragment)
237	0	[5 IU – H]^−^ (nucleobase; N-glycosidic bond rupture)
127	0	I^–^ (nucleobase)
110	0; 1.2	[5IUrd – I – R]^−^ (nucleobase)
94 (237–127–16)	0	[5IUrd – R – I – O]^−^ (nucleobase)
81 (110– 29)	0.2	[5IUrd – R – I – COH]^−^ (nucleobase)
71 (133–62)	0	[R – C_2_O_2_H_6_]^−^ (ribose)
46	0	C_2_OH_6_^–^ (ribose)
42	0; 4.4; 6.6	OCN^–^ (nucleobase)
26	0; 1.8	CN^–^ (nucleobase)
16	7.1; 9.3	O^–^ (either nucleobase or ribose)

a 5IUrd (370 u) = [5 IU – H] (237 u) + [ribose – OH] (133 u) (where 5IUrd = C_9_H_11_IN_2_O_6_, 5 IU = C_4_H_3_IN_2_O_2_, and ribose = C_5_H_10_O_5_). Here and throughout this work, R stands for [ribose – OH].

At low energy, it is well established that molecular dissociation is efficiently driven by dissociative electron attachment (DEA).^34^ In brief, a colliding electron is temporarily trapped by a molecule, forming a transient negative ion (TNI^#–^). This intermediate may decay into a negative ion, detected by mass spectrometry, and one or more neutral counterparts, provided that the dissociation occurs faster than electron autodetachment (i.e., within the survival time of the transient anion). The resulting anion signal reflects a convolution of the electron capture cross section at the resonance state and the survival probability of the transient anion.

At low incident electron energies, the cross section for TNI formation follows an E^–1/2^ energy dependence and can be substantial near 0 eV. Importantly, efficient formation of a stable anionic fragment at near-zero electron energy requires that the corresponding dissociation channel be exothermic—that is, the electron affinity (EA) of the fragment retaining the electron must exceed the bond dissociation energy (BDE) of the cleaved bond. This thermodynamic condition is critical for efficient DEA and underlies the enhanced cytotoxicity of 5IUrd compared to other halogenated nucleosides, as will be shown in the following sections.

For instance, the observation of I^–^, ([5 IU – H]^−^, and [(5IUrd – I – R)]^−^ anions indicates the concomitant production of the associated neutral radicals: [M – I]^•^, R^•^, and I^•^ plus R^•^, respectively. Shape resonance (i.e., capture of the extra electron in a usually unoccupied molecular orbital, MO^31^) or core-excited resonance (i.e., excitation of a core valence electron into some excited MO, concomitantly with the trapping of the excess electron by the molecular positive core^31^) are the possible mechanisms for the decay of TNI^#–^. In the case of the first mechanism, which typically occurs below the first electronically excited state of the precursor molecule (≲ 4 eV), the extra electron is likely trapped into a dissociative antibonding σ* MO or into π* decaying into σ* MO, as discussed for the nucleobases and ribose subunits^27,28,35,36^ as well as halogenated nucleotides.^25,37^ This mechanism is most likely responsible for the formation of most of the fragment anions shown in Figures 1 and 2. Core-excited resonances are observed at higher energies, i.e., the O^–^ anion (Figure 2). A resonance at 7.5 eV has been calculated not only for glucose, which partially implicates the σ* character of the C–O bonding in the molecule,^38^ but also for the canonical nucleobase uracil.^39^

Three dissociation channels producing the fragment anions and their associated neutral counterpart deserve a particular discussion: (1) I^–^/[M – I]^•^ (*m*/*z* 127), (2) [5 IU – H] ^–^/R^•^ (*m*/*z* 237), and (3) [(5IUrd – I – R] ^–^/(I^•^ + R^•^) (*m*/*z* 110). The first dissociation channel results from the cleavage of the C–I bond. The second channel involves the cleavage of the N-glycosidic bond. The third channel, in addition to the rupture of the C–I bond at the nucleobase moiety, also includes the cleavage of the N-glycosidic bond. The energetics of bond dissociation is controlled by the electron affinity of the negative ion and the bond dissociation energy. As the values of EA­(I) and C–I bond energy are found to be 3.06 eV^40^ and 2.69 eV,^41^ respectively, the dissociation channel (1) is already exothermic at room temperature. The N-glycosidic bond rupture, dissociation channel (2), is also exothermic or at least thermo-neutral at room temperature, considering the electron affinity of the nucleobase radical (at the N1 site) and the C–N bond energy (i.e., 3.6–3.8 eV^42,43^ and 3.0–3.6 eV,^44^ respectively). The dissociation channel (3), producing the *m*/*z* 110 fragment anion, must arise from the initial attachment of the extra electron to precursor 5IUrd, followed by expelling both the iodine and the R radicals. For the other fragment anions, only O^–^, CN^–^ and OCN^–^ are unambiguously assessed to the fragmentation of nucleobase moiety. The high electron affinity of the CN and the OCN radicals (i.e., 3.862 and 3.609 eV, respectively (**39**)) drive the nucleobase ring fragmentation. Table 1 tentatively assigns the observed anions to the corresponding complex dissociation channels.

The fragmentation of 5IUrd induced by low energy electrons differs from that of thymidine,^25,45^ 5-bromouridine (5BrUrd),^46^ and 5-fluorouridine (5FUrd) (**30**). In thymidine, cleavage of the N-glycosidic bond results in nearly equal formation of [T – H]^−^/R^•^ and R^–^/[T – H]^•^. Dissociation of 5BrUrd predominantly produces Br^–^/[5BrUrd – Br]^•^ and [5BrU – H]^−^/R^•^ in a ratio of 94% to 6%, respectively. Finally, in the case of 5FUrd, three dominant dissociation channels have been reported: [5FUrd – H]^−^/H^•^, [5FU – H]^−^/R^•^ and HCO_2_^–^ from ribose fragmentation, occurring at approximately 21%, 43% and 36%, respectively. For 5IUrd, the I^–^/[M – I]^•^ channel accounts for nearly 99% of all accessible fragmentation pathways (Figures 1 and 2), while cleavage of the N-glycosidic bond appears to be insignificant in comparison to the other studied halo-substituted uridine nucleosides and thymidine. Molecular dissociation can be quantified by evaluating the DEA cross section for each fragmentation channel. In the first approximation, the number of measured ions in our experiment, regardless of their nature, *N*_ions_, can be estimated as *N*_ions_ = ε.*N*_e_.(*N*_mol_/*V*)­σ.*L*, where ε is the detection efficiency (assuming the same for all ions), *N*_e_ is the number of electrons (or current), *N*_mol_/*V* is the density of the target molecule (proportional to the injected gas pressure), σ is the ion production cross section, and *L* is the collision length. Thus, the relative fragmentation cross section can be estimated by comparing the integrated yield of the negative ions with that of the calibration gas, SF_6_^–^, according to the ratio: σ_ion_/σ_SF6_. Knowing the cross section for the formation of the SF_6_^–^ anion at 420 K (ca., ∼9 × 10^–14^ cm^2^ within 5–10%),^47,48^ the approximate DEA cross section for the production of the I^–^ anion, and consequently [5IUrd – I]^•^ neutral radical, can be estimated to be (2.7 ± 1.9) × 10^–14^ cm^2^, with a 95% confidence level. This value represents the average of five independent experiments conducted on consecutive days. In comparison, the cross section for N-glycosidic bond cleavage induced by low energy electrons has been evaluated to be 4 × 10^–17^ cm^2^ (430 K) for thymidine^25^ and 6.8 × 10^–15^ cm^2^ for 5FUrd.^30^ It should be noted that the cross section value determined in this work at 428–430 K may typically decrease by about 1 order of magnitude at room temperature, as previously observed for thymine,^49^ thiouracil,^50^ and thiothymine.^51^

Electron impact on gaseous 5-iodouridine produces the I^–^ anion in association with its neutral [5IUrd – I]^•^ radical counterpart, which constitutes, by far, the predominant dissociation channel (∼99%). This result resembles previous studies of 5BrUrd, where the formation of the halogen Br^–^ anion was also reported as the dominant dissociation channel, accounting for approximately 94%. For both 5IUrd and 5BrUrd, the dissociation channel leading to halogen anion formation is exothermic, and thus accessible by 0 eV electrons already at room temperature. In contrast, for 5FUrd and 5CH_3_Urd (thymidine), the production of F^–^ or CH_3_^–^ is not accessible at 0 eV, and fragmentation is instead dominated by N-glycosidic bond cleavage (∼50%). By considering the fragmentation cross sections, it is noteworthy that the damage to 5IUrd was found to be greater than that observed for thymidine and 5-fluorouridine by about 700 and 4 times, respectively. This result is not surprising and to some extent reflects the reduced surviving fraction observed in radiation treatments of human bone marrow or bladder cancer cells^52^ with and without 5IUrd substitution. It is likely that such LEE-induced fragmentations, observed here in gas-phase experiments at 428–430 K, also occur in more realistic environments containing water, as local temperatures in the vicinity of the ionizing track may rise dramatically above 400 K.^53^

The abstraction of the halogen anion from the halogenated nucleobase produces the reactive uracil-yl [5U-yl]^•^ radical,^18^ which is widely recognized as a precursor to DNA strand breaks:^54–56^ [5U-yl]^•^ may undergo hydrogen abstraction either from the adjacent deoxyribose group or from structural water molecules located in DNA grooves, generating highly reactive OH^•^ radicals and ultimately resulting in DNA strand breaks.^52^ The present findings support the high efficiency of 5IUrd as a radiosensitizer in radiotherapy, particularly since its cellular incorporation is now facilitated by the pro-drug ropidoxuridine. This efficiency may be further enhanced through synergistic combinations with clinically accepted agents such as cisplatin (e.g., Lipoplatin )^57–59^ or gold nanoparticles,^60,61^ as both gold and platinum atoms can act as additional sources of secondary low-energy electrons under high-energy irradiation.^62,63^
